# Symmetry breaking, germ layer specification and axial organisation in aggregates of mouse embryonic stem cells

**DOI:** 10.1242/dev.113001

**Published:** 2014-11-15

**Authors:** Susanne C. van den Brink, Peter Baillie-Johnson, Tina Balayo, Anna-Katerina Hadjantonakis, Sonja Nowotschin, David A. Turner, Alfonso Martinez Arias

**Affiliations:** 1 Department of Genetics, University of Cambridge, Cambridge CB2 3EH, UK; 2 Developmental Biology Program, Sloan-Kettering Institute, New York, NY 10065, USA

**Keywords:** Mouse, Gastrulation, Self-organisation, Symmetry breaking, Polarisation, Axial elongation, Endoderm, Mesoderm, Neural ectoderm, Pattern formation, Live cell imaging

## Abstract

Mouse embryonic stem cells (mESCs) are clonal populations derived from preimplantation mouse embryos that can be propagated *in vitro* and, when placed into blastocysts, contribute to all tissues of the embryo and integrate into the normal morphogenetic processes, i.e. they are pluripotent. However, although they can be steered to differentiate *in vitro* into all cell types of the organism, they cannot organise themselves into structures that resemble embryos. When aggregated into embryoid bodies they develop disorganised masses of different cell types with little spatial coherence. An exception to this rule is the emergence of retinas and anterior cortex-like structures under minimal culture conditions. These structures emerge from the cultures without any axial organisation. Here, we report that small aggregates of mESCs, of about 300 cells, self-organise into polarised structures that exhibit collective behaviours reminiscent of those that cells exhibit in early mouse embryos, including symmetry breaking, axial organisation, germ layer specification and cell behaviour, as well as axis elongation. The responses are signal specific and uncouple processes that in the embryo are tightly associated, such as specification of the anteroposterior axis and anterior neural development, or endoderm specification and axial elongation. We discuss the meaning and implications of these observations and the potential uses of these structures which, because of their behaviour, we suggest to call ‘gastruloids’.

## INTRODUCTION

The emergence of asymmetries within a mass of otherwise equivalent cells is the starting event in the development and patterning of all embryos, and results in the establishment of a coordinate system that cells use as a reference to generate the main axes of an organism. In animal embryos the axial organisation acts as a reference for the process of gastrulation, a choreographed sequence of cell movements that transforms an often hollow epithelium into a three-layered structure endowed with a blueprint for the organism: a head at the anterior pole and, in vertebrates, the ectoderm, that will give rise to the nervous system on the dorsal side and the endoderm and the mesoderm on the ventral side. The process of gastrulation is driven by coordinated movements of groups of cells that interpret the global coordinate system of the embryo and give rise to the endoderm and the mesoderm (Norris et al., 2002; Nowotschin and Hadjantonakis, 2010; Ramkumar and Anderson, 2011; Solnica-Krezel and Sepich, 2012). Although the outcome of gastrulation is highly conserved, the mechanics of the process varies, even within a phylum (Keller et al., 2003). Thus, within the chordates, anamniotes such as amphibians utilise the convergence of a ring of cells into a small opening, or blastopore, through which mesoderm and endoderm invaginate and perform directional movements, whereas in amniotes, such as chicken, mouse and the primates, the invaginating cells move within a different geometry and configure a dynamic groove of cells called the primitive streak (PS) that acts as the source of the endoderm and mesoderm (reviewed by Keller et al., 2003; Solnica-Krezel and Sepich, 2012; Tam and Loebel, 2007). In all embryos there is a close relationship between the process of gastrulation and the establishment of axial structures, as experiments and mutants that disturb the establishment of the axis lead to profound alterations in the specification and movement of the endodermal and mesodermal precursors (Huelsken et al., 2000; Medina et al., 1997; Morkel et al., 2003). These defects have secondary effects on the development of the nervous system.

Studies with mouse and human embryonic stem cells (ESCs) have shown that culture of 3D aggregates termed embryoid bodies (EBs) leads to the formation of rudiments of tissues and organs without the context of an embryo (Desbaillets et al., 2000; Höpfl et al., 2004). Recently, EBs have been steered to differentiate into eye cups and anterior neural cortical structures (Eiraku et al., 2011; Lancaster et al., 2013; Nakano et al., 2012; Sasai, 2013; Sasai et al., 2012), a remarkable feat as these structures emerge without a recognisable reference coordinate system. An explanation for this observation might lie in the intrinsic tendency of mouse ESCs (mESCs) to develop anterior neural fates (Tropepe et al., 2001; Turner et al., 2014c; Watanabe et al., 2005; Wataya et al., 2008). In contrast to these observations, there are no reports of the emergence of axial structures in EBs, even though in culture it is possible to obtain progenitor cells for mesodermal and endodermal structures (Gadue et al., 2006; Kouskoff et al., 2005; Kubo et al., 2004) that exhibit some of the morphogenetic properties of the embryo (Turner et al., 2014b), and signalling can elicit a degree of polarised gene expression in EBs (ten Berge et al., 2008). One exception was reported in a study of P19 embryo carcinoma (EC) cells. Under differentiation conditions, EBs made from these cells can organise themselves into polarised and extending structures resembling gastrulating embryos (Marikawa et al., 2009). Such large-scale organisation has not been described in ESCs.

Here we show that small aggregates of mESCs undergo a symmetry-breaking event in culture and that, under conditions that promote the formation of mesendoderm in embryos, they exhibit polarised expression of the endoderm marker Sox17 (Kanai-Azuma et al., 2002) and FoxA2 (Monaghan et al., 1993; Sasaki and Hogan, 1993) and of the PS and early mesoderm marker brachyury (Bra, or T) (Herrmann, 1991). Over time, Bra expression becomes restricted to a small population of cells at a tip of the aggregate, which acts as a source of cells that express *Tbx6*, a mesoderm gene (Chapman et al., 1996), and these cells are extruded from the main body of the aggregate in a process that is reminiscent of some of the movements of gastrulation. For this reason, we call these aggregates ‘gastruloids’ and show that, although for the most part they are autonomous in their development, the culture conditions influence the cell types that develop within them. We compare the behaviour of these aggregates with that of embryos and discuss their potential as a new experimental system with which to study mechanisms of early mammalian development.

## RESULTS

### Symmetry breaking in differentiating EBs

The observation that P19 EC cells are able to form polarised, elongated structures during differentiation (Marikawa et al., 2009) prompted us to seek culture conditions in which EBs derived from mESCs would develop similar structures. When cells were placed in a serum and LIF hanging drop culture, cells formed aggregates and, after removal of LIF, a small proportion changed their morphology to an ovoid appearance, although any further suggestion of elongation was never apparent (data not shown). In order to stimulate the emergence of PS features, we used culture conditions that steer the cells towards this fate in adherent culture (Gadue et al., 2006; Turner et al., 2014b,c) and exposed EBs of different sizes to N2B27 for 2 days followed by continuous treatment with both activin A (Act) and CHIR99021 (Chi), a Wnt/β-catenin signalling agonist (Act/Chi conditions) (supplementary material Fig. S1A; see Materials and Methods for details).

The initial EBs contained ∼800-1000 cells and during the first phase of aggregate formation in N2B27 we noticed that, in contrast to cells hanging in serum and LIF where only one aggregate was formed per drop (supplementary material Fig. S1D), cells in N2B27 formed multiple aggregates of variable sizes per drop (supplementary material Fig. S1C). Following the change of medium into Act/Chi conditions, the aggregates dispersed and over time we observed an increasing number of aggregates adopting a shape that differed from their original spherical appearance (supplementary material Fig. S1E-F‴); some displayed an ovoid shape (supplementary material Fig. S1E), resembling what has been described previously for EBs when β-catenin is activated (see Figure 4B in ten Berge et al., 2008). However, we also observed clear elongation in a few aggregates (supplementary material Fig. S1E) and, by the fourth day in Act/Chi, a median of 30% of aggregates exhibited a polarised, elongated morphology. The reduction in the proportion of cells displaying elongated aggregates at later time points reflected an increase in cells displaying a differentiated phenotype in addition to an increase in an apoptotic appearance (data not shown). The aggregates needed to be in suspension for shape changes to occur (Baillie-Johnson et al., 2014).

These results indicate that it is possible to elicit symmetry breaking and polarisation in aggregates of ESCs.

### The effect of signals and aggregate size on polarisation

The heterogeneous response of the EBs to our experimental treatment could be due to several factors and we decided to focus on three that we deemed to be most influential in the outcome of the experiment: (1) the composition of the culture medium; (2) the timing of exposure; and (3) the initial size of the aggregates. In these experiments we moved from culturing the aggregates in hanging drop to 96-well plates, as this allowed us to arrange for one, and only one, aggregate to develop in each well, minimising the possibility of fusions (for details see Baillie-Johnson et al., 2014). The aggregates were first placed in N2B27 for 2 days and assayed on the fifth day of culture.

**Fig. 1.**
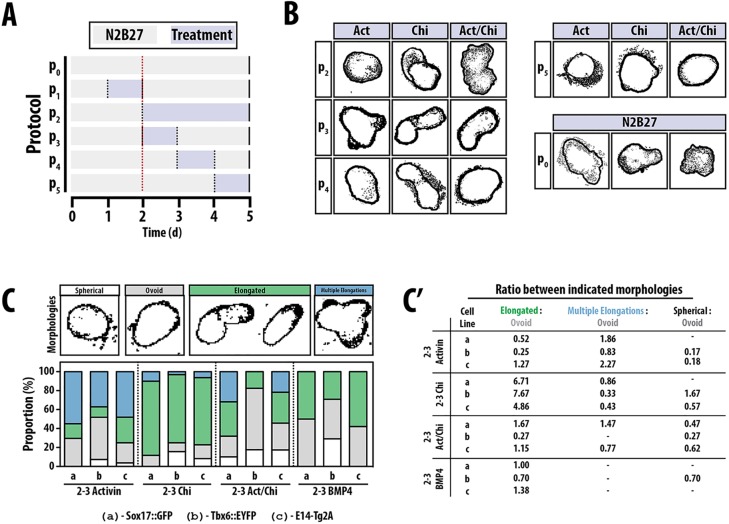
**Comparative analysis of the effect of exposure time and signalling on aggregate formation.** (A) The stimulation protocol. The vertical black dotted lines indicate medium changes, and the red vertical line corresponds to the beginning of day 2. Aggregates were cultured in N2B27 (grey shading) continuously (P_0_), or treated with continuous (P_2_) or 24 h pulses (P_1,3,4,5_) of Act, Chi or Act/Chi (blue shading) before being returned to N2B27. Data for P1 not shown. (B) Cartoon renderings (see Materials and Methods; unprocessed images are shown in supplementary material Fig. S2) of typical aggregate morphologies on day 5 following the conditions shown in A; images are not to scale. Maximum elongation was observed following pulsed treatment within the day 2-3 time frame (P_3_). (C,C′) Comparison of aggregate morphologies following a 24 h pulse on days 2-3 of Act, Chi, Act/Chi and BMP4 for three different cell types: (a) Sox17::GFP, (b) TBX6::EYFP and (c) wild-type E14-Tg2A. (C) Aggregates were scored based on whether they were spherical (white), contained a single outgrowth (ovoid, grey), showed overt elongation (green) or had multiple protrusions (blue). Examples of these aggregate morphologies are shown in the form of cartoon renderings, processed as described above. (C′) These data are also represented as ratios between the indicated morphologies and the proportion of aggregates with ovoid appearance. Note how transient exposure to Chi results in a much higher ratio of elongated to ovoid morphologies. The number of Sox17::GFP, Tbx6::EYFP and E14-Tg2A aggregates for each condition (C,C′) are as follows (respectively): Act: 71, 27, 52; Chi: 60, 32, 48; Act/Chi: 69, 17, 46; BMP4: 64, 24, 38.

If, after the initial 2 days in N2B27, the aggregates are left in this medium then we observe a range of morphologies, with 20-30% exhibiting some polarisation. When signals (Act, Chi and BMP) are applied continuously from the third day of differentiation, the response is signal specific: in Act/Chi many of the aggregates exhibit a weak elongation, whereas continuous exposure to Act alone elicits a variable number of short protrusions or invaginations per aggregate; on its own Chi triggers a smaller number of longer and broader protrusions (Fig. 1A,B, protocol P_2_). In all cases there is a variability in the response of the aggregate to a particular culture condition that changes with the cell line, although the structure that emerges is specific and recognisable for each of the signals (Fig. 1B).

Using Act and Chi in combination (Act/Chi) or individually, we next tested the effect of changing the timing of exposure to signals (Fig. 1A,B, protocols P_3-5_). We began by restricting the exposure of these signals from differentiation days 2-5 (Fig. 1A, protocol P_2_) to days 2-3, 3-4 and 4-5 (Fig. 1A, protocols P_3_, P_4_ and P_5_, respectively), and returning the aggregates to N2B27 for the duration of the timecourse. Limiting the exposure to the third day (48-72 h) triggered the most reproducible response, with greater than 70% of the aggregates undergoing similar morphological changes (Fig. 1C,C′). Under these conditions, Act alone produces a number of small, broad protrusions and invaginations from a large oval. Addition of Chi to the Act reduces the number of invaginations and, in many instances, elicits a single elongation of ∼40-60 µm that is attached to a broad mesenchymal-like structure at the distal end of the aggregate (Fig. 1C,C′). On its own a short exposure to Chi consistently elicits a single elongation without clear protrusions or invaginations. Exposure to the different signals limited to either the second or the fourth day of culture produced more variable responses, and many aggregates that did not respond (Fig. 1). These results suggest that, during the third day of differentiation, ESCs are in a competent state to efficiently interpret signals in the medium.

**Fig. 2.**
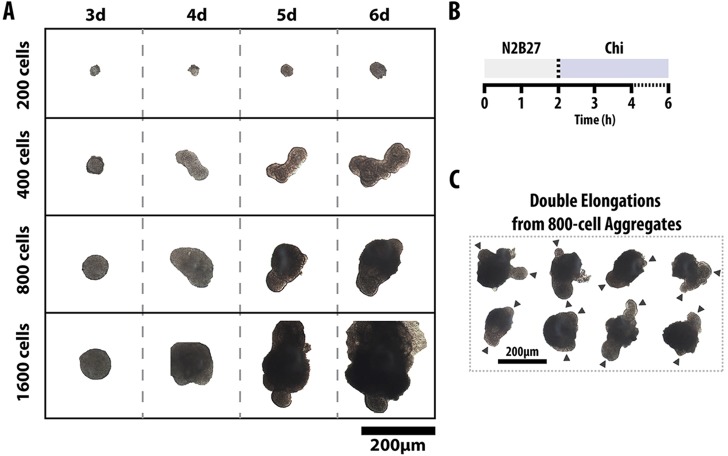
**Effect of initial cell density on the elongation of aggregates.** (A) Aggregates formed from increasing numbers of cells (200-1600 cells) as indicated were exposed to Chi for the duration of the experiment (at least six aggregates per condition). (B) Aggregates with an initial size of between 400 and 800 cells showed elongation. (C) Aggregates with 800 cells tended to exhibit multiple elongations (arrowheads).

As exposure to Chi from the third day elicited a simple and consistent response in the form of an elongation, we used this experimental condition as the basis to analyse the effect of the initial size of the aggregates on their polarisation (Fig. 2; supplementary material Fig. S2A-D). Starting with different numbers of cells revealed that only small aggregates, of ∼300±100 cells, exhibit the elongation effectively. Smaller aggregates (<200 cells) either grow or remain small and exhibit slow growth; when they grow, they tend to produce the elongation. Larger aggregates (>600 cells) grow in the disorganised and symmetrical manner that is regularly reported for EBs.

These results indicate that EBs configured from mESCs are capable of elongation in the manner that has been described for P19 EC cells (Marikawa et al., 2009) and that this behaviour is associated with specific culture conditions.

### Spatial and temporal patterns of gene expression in polarising EBs

**Fig. 3.**
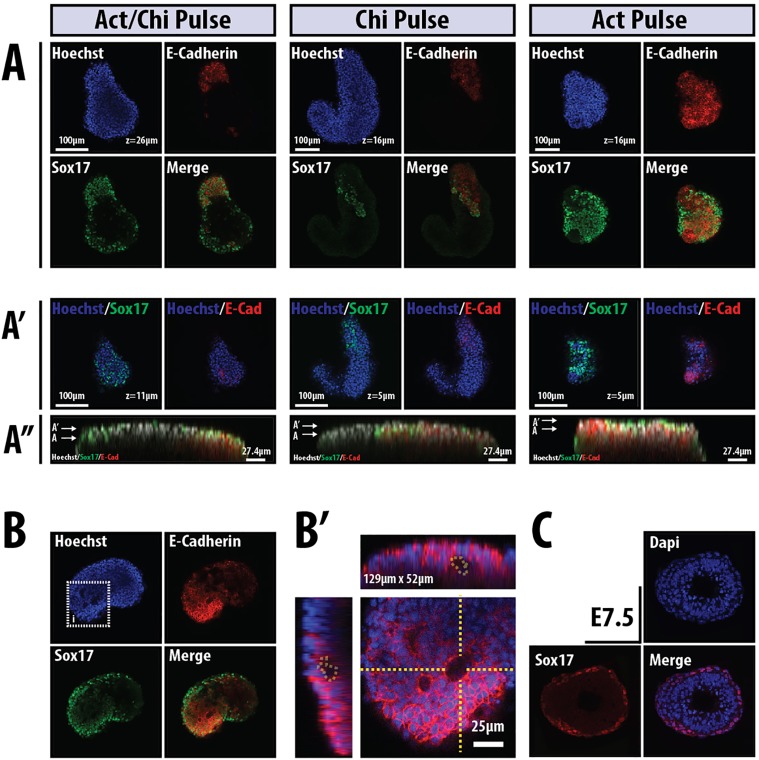
**Polarisation, patterning and gene expression in aggregates.** (A,A′) Two single sections through GPI-GFP mESC aggregates exposed to N2B27 for 5 days with a 24 h pulse of either Act (*n*=10), Chi (*n*=5), or Act/Chi (*n*=14) between 48 and 72 h and imaged by confocal microscopy (GPI-GFP channel not shown). The expression of the indicated markers on the surface of the aggregates is shown in A′, with the corresponding orthogonal view through the aggregate in A″. The arrows in A″ indicate the *z*-section shown in A and A′. Note how the expression of Sox17 is localised to the surface of the aggregate. (B,B′) A representative aggregate from GPI-GFP mESCs exposed to Act between 48 and 72 h was imaged at the end of the treatment after being fixed and stained for E-cadherin and Sox17 (B); the boxed region is enlarged to show E-cadherin (B′). Note the depressions that are associated with Sox17 expression and high levels of E-cadherin. (C) Section through an E7.5 embryo stained for Sox17 and with DAPI.

**Fig. 4.**
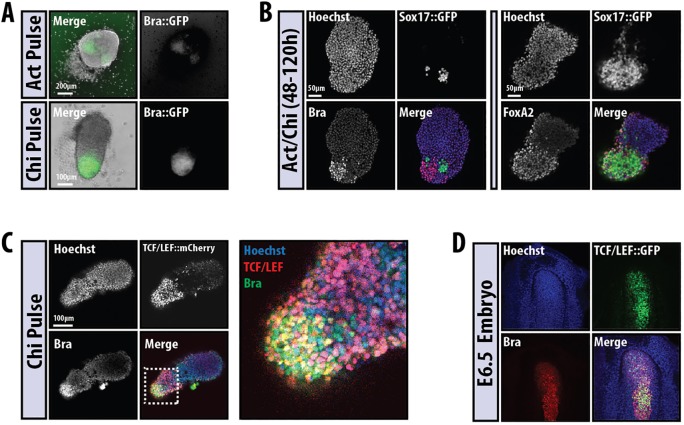
**Polarised expression of Bra in response to Wnt signalling.** (A) Bra::GFP cells exposed to a 24 h pulse of Act or Chi between 48 and 72 h and imaged at 120 h (*n*>3). (B) Aggregates of a Sox17::GFP cell line treated with sustained Act/Chi and stained on day 4 for either Bra (left, *n*>10) or FoxA2 (right, *n*=13). (C,D) The β-catenin transcriptional reporter line TCF/LEF::mCherry treated with (C) Chi in culture (as in A, *n*=11) and compared with its expression in the PS of an E6.5 embryo (D), both stained for Bra. These conditions not only produce elongations of the aggregates, but also result in polarised gene expression.

The elongated aggregates resemble structures that have been described in sea urchin and amphibian embryos (Holtfreter, 1933; Horstadius, 1939; Ishihara et al., 1982; Keller and Danilchik, 1988) or when animal caps from *Xenopus* embryos are exposed to Activin (Green et al., 2004; Ninomiya et al., 2004; Symes and Smith, 1987). These comparisons suggested to us that the elongated bodies might be recapitulating some of the early events associated with gastrulation. If this were the case, the cells involved in generating the protrusions might represent mesendodermal tissue. To address this and exclude the possibility that the protrusion is simply a mechanical response to the size and shape of the aggregates without a specific fate (i.e. that there is no correspondence between structure and fate), we analysed the expression of genes associated with early differentiation in culture and in embryos (Fig. 3). To begin with we analysed the expression of Sox17 (Figs 3 and 4), a marker of primitive and definitive endoderm (Kanai-Azuma et al., 2002), and of Bra (Fig. 4), a gene associated with the specification of endoderm and mesoderm in the PS (Herrmann, 1991), using fluorescent reporter ES cell lines for both genes (Fehling et al., 2003; Niakan et al., 2010). Aggregate formation and staining with Sox17 and Bra antibodies confirmed that both lines are faithful reporters of the expression of the genes (supplementary material Fig. S3) (Turner et al., 2014b).

Following a transient exposure during day 3 to either Act or Act/Chi we observe expression of Sox17::GFP mostly in clusters of cells which, in the presence of Act/Chi, tend to lie within the elongating region (Fig. 3A). Confocal optical sections through the aggregates revealed them to be multi-layered structures (Fig. 3A′,A″) with a number of aggregates displaying internal cavities and localised indentations or pits on their surface (Fig. 3B,B′; supplementary material Movie 1). Sox17 expression is, for the most part, restricted to the external cells and is associated with E-cadherin (Fig. 3A), as it is in the embryo. The amount of Sox17 expression increases with the time of exposure and requires Act, as exposure to Chi alone reduces the levels of expression and the number of expressing cells (Fig. 3A). In all cases, Sox17-expressing cells tend to invaginate, retain E-cadherin expression and form vesicles near the surface of the aggregate (Fig. 3B; see also Fig. 7F).

Further analysis of aggregates exposed to Act and, particularly, to Act/Chi revealed expression of Bra (Fig. 4A,B), Sox17 (Fig. 3 and Fig. 4B) and FoxA2 (Fig. 4B), which are all associated with the PS, localised towards the extending tip of the aggregate; Bra and Sox17 are expressed in a mutually exclusive pattern (Fig. 4B). Whereas the expression of Sox17 coincides with that of FoxA2 (Fig. 4B), that of Bra correlates with a high level of β-catenin transcriptional activity as demonstrated in a TCF/LEF::GFP (TLG) reporter cell line (Fig. 4C). These patterns of expression are reminiscent of those in gastrulating embryos, in which Bra and Wnt/β-catenin signalling can be observed in the PS (Fig. 4D) (Ferrer-Vaquer et al., 2010). Taken together, these observations suggest that the aggregates formed from mESCs undergo morphogenetic movements that resemble the early stages of gastrulation.

### Imaging symmetry-breaking events in real time in the aggregates

**Fig. 5.**
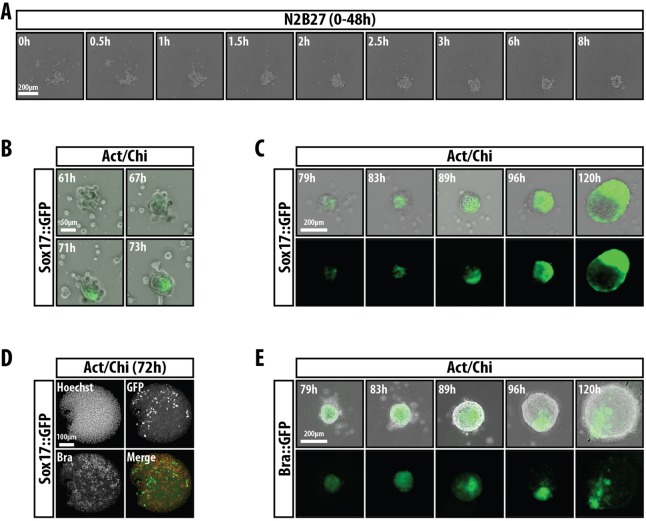
**Emergence of polarised gene expression in aggregates.** (A) Stills from live cell imaging of mESCs in suspension in N2B27 showing aggregate formation within the first 8 h. (B,C) Emergence and progression of Sox17::GFP following addition of secondary Act/Chi medium. (D) Early stages of Sox17::GFP and Bra expression. Initially, Bra and Sox17::GFP are heterogeneously expressed before polarisation occurs. (E) Live imaging of Bra::GFP mESCs following addition of Act/Chi. Every cell initially expresses Bra before downregulation in regions that will not form the elongation. A, B, C and E are from supplementary material Movies 1-4, respectively. Data are representative from at least two experiments.

In order to monitor the emergence of the polarised expression of Sox17 and Bra, we performed live cell microscopy on E14-Tg2A, Sox17::GFP and Bra::GFP transcriptional reporter cell lines. First, we imaged the formation of the aggregates from a cell suspension of mESCs in N2B27 for 48 h (Fig. 5A; supplementary material Movie 2). Individual cells or clusters containing small numbers of cells were found to coalesce into larger aggregates due to a combination of the spatial constraints of the round-bottomed culture wells and active cell movement towards the aggregate (Fig. 5A; supplementary material Movie 2). During this time we do not observe expression of visceral endoderm (VE) markers, such as Gata6 (not shown) or Sox17, suggesting that the aggregates are composed exclusively of embryonic tissues. We then focused on the Act/Chi conditions, recording the emergence of the fluorescence from the time of transfer from the N2B27 medium into Act/Chi (Fig. 5B,D) and then at later stages when the aggregates were more advanced (Fig. 5C,E; supplementary material Movies 2-4). In both cases, we observe the development of polarised gene expression over time, but the patterns are different for each of the genes.

In the case of Sox17 (Fig. 5B,C), after ∼32 h in Act/Chi we observe an initial pattern of scattered cells expressing the reporter intermingled with Bra-expressing cells (Fig. 5B,D; supplementary material Movies 3 and 4). The videos suggest that the definitive, polarised expression pattern is established from the aggregation of Sox17-expressing cells on one side of the aggregate, which then proliferate and at 96 h can be seen to be associated with the elongation. Clusters of Sox17-expressing cells can be seen to move inside the aggregate, which would be consistent with the invaginations described above (Fig. 4B,C). In the case of Bra (Fig. 5D,E; supplementary material Movies 5 and 7), Bra::GFP was initially expressed transiently across the whole aggregate (79 h in secondary medium; Fig. 5E) before becoming restricted to a small region (Fig. 4E, Fig. 5D,E). Downregulation of the reporter in other regions of the aggregate appeared to be undertaken by individual cells not within the region of high expression. As time progressed, the aggregate increased in size and maintained the expression of Bra::GFP within one region (Fig. 5E; supplementary material Movie 5). These results suggest that symmetry breaking and polarisation of gene expression are a feature of these aggregates elicited by different signals.

### Signalling and pattern formation during aggregate differentiation

**Fig. 6.**
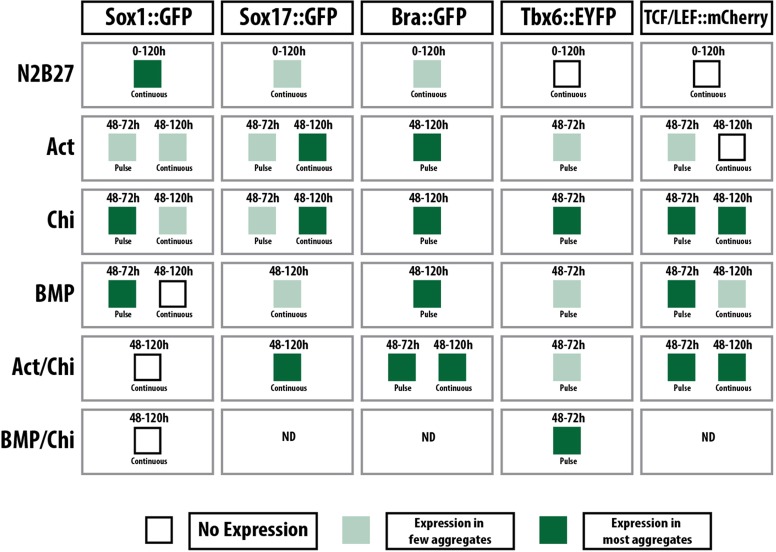
**Qualitative summary of the tissue-specific response of aggregates to different signalling environments.** Reporter lines were used for neural (Sox1::GFP), endoderm (Sox17::GFP), mesendoderm (Bra::GFP) and paraxial mesoderm (TBX6::EYFP) as well as for Wnt signalling (TCF/LEF::mCherry). Aggregates from the different lines were treated as indicated and the results in terms of expression levels within the population as a whole are summarised by the colour intensity within each square. Representative examples are shown in Fig. 7. ND, not determined.

In the early postimplantation epiblast, cell fate assignments are triggered by interactions between BMP, Nodal, Wnt signalling and their antagonists, and lead to the partitioning of the embryo into anterior neuroectodermal and posterior mesendodermal populations (Arnold and Robertson, 2009; Pfister et al., 2007; Tam and Loebel, 2007). To expand our studies beyond mesendoderm, we used a Sox1::GFP reporter ES cell line to monitor neural development (Ying et al., 2003), and a TBX6::EYFP line to follow the emergence of mesoderm (see Materials and Methods). We also included BMP (see also Fig. 1C) in the repertoire of signals, as it plays a role in the early stages of embryonic patterning (Arnold and Robertson, 2009; Tam and Loebel, 2007). In these experiments, aggregates were exposed to the signals either during the third day of differentiation and then returned to N2B27 for a further 2 days, or for the last 3 days of the experiment (as summarised in Fig. 6).

**Fig. 7.**
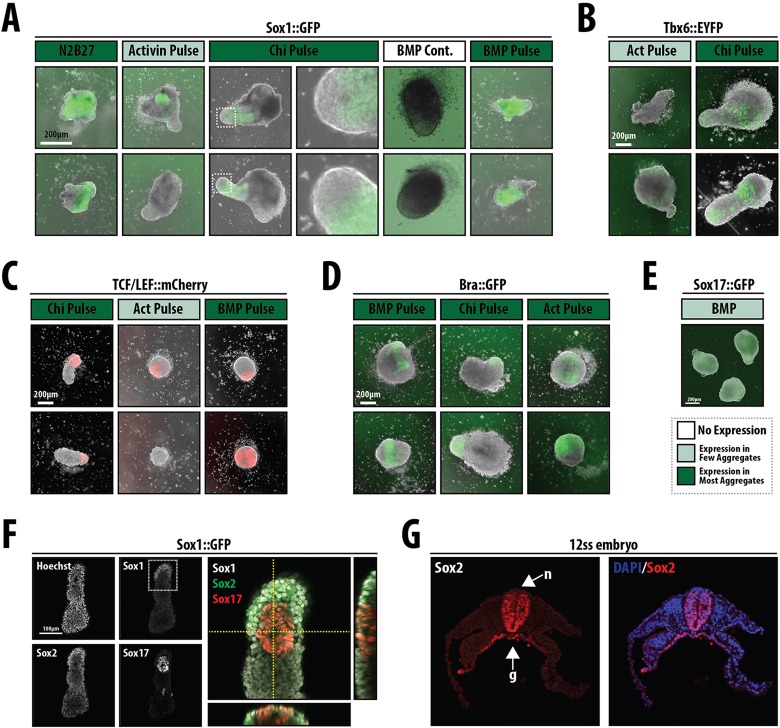
**Gene and tissue-specific response of aggregates to different signalling environments.** (A-E) Representative examples from the summary in Fig. 6. Sox1::GFP (A, *n*=16, 14, 16, 24, 16 per labelled condition, respectively), TBX6::EYFP (B, *n*=11 or 10 per labelled condition, respectively), TCF/LEF::mCherry (C, *n*=4 per condition), Bra::GFP (D, *n*=9, 11 or 11 per labelled condition, respectively) and Sox17::GFP (E, *n*>3) mESCs were treated as indicated. The boxed region in the Chi pulse image (A) is enlarged section to the right to show a region within the tip of the aggregate that is negative for Sox1::GFP. Compare with the expression pattern of Bra and Sox17::GFP and Wnt activity from Figs 3 and 4. Note that the expression of the reporters is associated with specific morphogenetic events; two examples of each are given. The colour coding of each treatment label corresponds to that used in Fig. 6. (F,G) Aggregates of Sox1::GFP mESCs following a pulse of Chi on day 3 were stained for GFP (Sox1), Sox2 and Sox17. The boxed region is magnified to the right and also shows orthogonal views. Sox1 at the tip of the aggregate is co-expressed with high levels of Sox2, whereas Sox2 levels decrease in regions high for the endoderm marker Sox17. (G) Section through a 12-somite stage embryo stained for Sox2 and with DAPI. Note how Sox2 is expressed in the neural tissue (n) and in the gut (g), similar to the expression pattern seen in F.

When cells are left in N2B27, for the most part they do not undergo any specific morphogenetic process and express Sox1::GFP throughout the aggregate (Fig. 6 and Fig. 7A), although in ∼10% of cases we observe some polarised Bra expression (not shown). The pattern of Sox1::GFP expression does not change when inhibitors of Nodal/Activin (SB43) or MEK (PD03) are added to the medium from day 3 (not shown) and is consistent with the observation that mESCs placed in N2B27 will develop, mainly, as neural precursors (Ying et al., 2003). Also consistent with known inputs of signalling on neural development (Andoniadou and Martinez-Barbera, 2013; Turner et al., 2014c), Sox1::GFP expression was suppressed by exposure to Act, although a few foci of expression remained in some aggregates (Fig. 6 and Fig. 7A). The response of genes associated with endoderm (*Sox17*) and mesoderm (*Tbx6*) to the different signals is summarised in Fig. 6 (examples of expression patterns are shown in Fig. 7). The pattern of responses mirrors that of embryos to the same signals (Figs 6 and 7). For example, Act suppresses mesoderm and promotes endoderm, whereas BMP promotes mostly mesoderm and Chi is able to elicit all germ layers (Fig. 6). In all cases the different cell types emerge as continuous and polarised groups of expressing cells: a pulse of Chi leads to an increase in TBX6::EYFP expression (Fig. 7B) and polarised β-catenin transcriptional activity (Fig. 7C) and Bra::GFP expression (Fig. 7D). We also observe interactions between the different signals; thus, BMP appears to quench the effects of Chi on Sox1 expression, and Act suppresses the effects of BMP on Tbx6 expression (not shown). Prolonged exposure to a signal or signal combination tends to increase the response in terms of expression but has a negative effect on polarisation of the expression (Fig. 6 and data not shown).

**Fig. 8.**
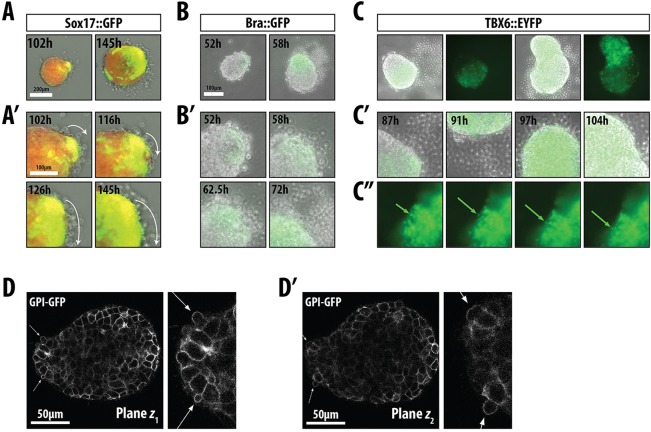
**Gastrulation-like movements in aggregates.** (A-C″) Cell extrusion and intra-aggregate movement in (A,A′) Sox17::GFP, (B,B′) Bra::GFP and (C-C″) TBX6::EYFP. Aggregates were treated as indicated. Images correspond to the indicated time points from the associated movies (see supplementary material Movies 5-8). Arrows in C″ indicate a single extruded cell from a second aggregate between 102 and 103 h (see supplementary material Movie 8); curved arrows in A′ indicate the direction of movement of the cells leaving the aggregate. (D,D′) Live imaging of GPI-GFP mESCs following treatment with Act/Chi, showing membrane blebbing at the elongated region of the aggregate. Two different *z* planes are shown. Arrows indicate the blebbing region (see supplementary material Movie 10).

The exposure to a pulse of Chi led to an elongation that, surprisingly, exhibits Sox1::GFP expression in the elongating cells (Fig. 7A), with Bra expression restricted to the tip of the elongate in a small region that does not express Sox1::GFP (Fig. 7A, Chi pulse insets; Fig. 7D). The elongated region exhibits a complex structure, with most of the cells expressing Sox2 and, often, Sox17 in vesicles that form near the surface and have lower levels of Sox2 (Fig. 7F); Sox1 expression is non-overlapping with that of Sox17. In the non-elongated region we observe low levels of Sox2 expression. This arrangement is reminiscent of the situation in the embryo, where the endoderm, which expresses Sox17 and Sox2 (Wood and Episkopou, 1999), lies underneath the developing nervous system (Fig. 7G). In addition, the aggregates express TBX6, which is usually associated with mesoderm formation (Chapman et al., 1996), and appear to recapitulate events associated with axial extension (see Turner et al., 2014a). The aggregates lack a notochord, which, in the embryo, lies between the nervous system and the gut. An important feature of the development of these aggregates is the timing of the events, which is reliable and reproducible from experiment to experiment: the Sox17 expression precedes and initially overlaps with Bra expression and the extrusion of cells, and Tbx6 expression follows a few hours later (see Fig. 8) (Turner et al., 2014a).

These results complement the morphological changes described above and are consistent with what is known about the early events in the embryo, namely the existence of a pre-proneural basal state in the epiblast with the mesendoderm being specified by BMP, Nodal/Act and Wnt signalling (Turner et al., 2014c). N2B27 appears to be a transitional medium in which cells can adopt a primary neural fate (Turner et al., 2014c). Furthermore, in the aggregates, as in the embryo, Act initiates endoderm development (Sox17) and BMP initiates mesoderm (Tbx6) development.

### Cell movement in polarised aggregates

When Sox17::GFP aggregates were cultured in Act/Chi for 120 h, we observed cells that were being extruded from a region adjacent to the primary focal point of reporter expression (Fig. 8A; supplementary material Movie 6). As time progressed, the frequency of this event increased and many more cells were seen to emerge from the same point. Close observation revealed two types of movement: Sox17-expressing cells appeared to move inside the aggregate, close to its wall, whereas others, not expressing Sox17, moved towards the outside (supplementary material Movie 6). Cell movements can also be observed after exposure to Chi and in other cell lines such as Bra::GFP (Fig. 7D and Fig. 8B; supplementary material Movie 7) and TBX6::EYFP (Fig. 7B and Fig. 8C; supplementary material Movies 8 and 9). Movies show that the extruded cells stem from the region of Bra::GFP expression (Fig. 7B) and that they express Tbx6 (Fig. 8C,C′; supplementary material Movies 8 and 9), suggesting that they are mesodermal. The extruded cells produce floating trails in the medium or attach to the main body of the aggregate (Fig. 7B and Fig. 8C′) and, when they attach, they maintain expression of Tbx6, suggesting that continuing Tbx6 expression requires some substrate that can only be provided by other cells. It appears as if the cells prefer to attach to the ‘anterior’ section of the aggregate, suggesting that there are differences between the two regions.

We do not observe filopodia or lamellipodia in the cells leaving the aggregate but observe the emergence of blebs (Fig. 8D,D′; supplementary material Movie 10), which have been associated with cell movements during gastrulation in zebrafish (Paluch and Raz, 2013).

## DISCUSSION

We have shown that under defined culture conditions aggregates of mESCs undergo processes that resemble the collective behaviours of cells in early mouse embryos: symmetry breaking, axial organisation, germ layer specification, gastrulation and axis elongation. This is surprising in light of the fact that EBs are commonly used in differentiation experiments and yet, with two exceptions, there have been no reports of similar behaviours. One of the exceptions is an account of elongation and polarised gene expression in aggregates of P19 EC cells exposed to serum (Marikawa et al., 2009). The second is a report of weak polarisation of Bra expression in EBs of mESCs exposed to agonists of Wnt signalling (ten Berge et al., 2008), which resembles the early stages of what we report here. It is possible that similar partial polarisation events occur at low frequency in EBs but are generally overlooked. We believe that the consistency and magnitude of the behaviour that we observe in our aggregates are founded, principally, in two aspects of our experimental protocol: the sequence of culture conditions that we use and the initial number of cells in the aggregate.

In our adherent cultures we had noticed that exposure of differentiating ESCs to N2B27 for 2 days results in a homogeneous response to external signals (Turner et al., 2014c). We reasoned that this treatment allows all cells in the culture to enter a state resembling the postimplantation epiblast, where they become competent to respond to signals (Sterneckert et al., 2010; Turner et al., 2014c), and it is for this reason that we used this protocol as the basis for our experiments. The second element in our protocol that differs from standard procedures concerns the number of cells in the initial aggregate, which appears to be a critical variable in the experiments. Aggregates above or below 300±100 cells (average aggregate diameter of 100 µm) will either not develop or do so into amorphous masses of cells, characteristic of the EB protocols in current use. This size of 300±100 cells is reminiscent of that of early postimplantation embryos and perhaps defines an optimal length scale for a unique outcome of the biochemical reactions that mediate symmetry breaking and polarisation. Experiments searching for conditions that mimic the emergence of the postimplantation epiblast from mESCs also find that the number of starting cells is a critical parameter of the process (Bedzhov and Zernicka-Goetz, 2014). A surprising conclusion from these observations that will need to be pursued is that early patterning events do not scale easily and this is in agreement with recent observations on the emergence of germ layers in micropatterns of human ESCs (Warmflash et al., 2014).

Our observations raise many questions about symmetry breaking in early embryos, the ability of cell ensembles to respond to signals and the different behaviour of mESCs in adherent and three-dimensional cultures. For reasons of space, here we shall focus on two specific issues concerning how the system that we have described informs our understanding of the mechanisms underlying early mammalian development; other issues will be discussed elsewhere (see Turner et al., 2014a).

### Symmetry breaking and axis specification

Our results reveal that, under appropriate culture conditions, aggregates of mESCs have an intrinsic ability for symmetry breaking and stable polarisation of gene expression. This pattern resembles events in the embryo at ∼E6.0 with some, perhaps informative, differences.

In the embryo, the initial localisation of the PS can be identified as a focus of Bra expression in the proximal posterior region of the embryo (Wilkinson et al., 1990) and its specification follows a sequence of events associated with the localisation of ligands for BMP, Nodal and Wnt signalling to the same region (reviewed by Arnold and Robertson, 2009; Pfister et al., 2007; Rossant and Tam, 2009; Tam and Loebel, 2007). This process requires first the specification and localisation of the anterior visceral endoderm (AVE) to the prospective anterior region of the conceptus, where it acts as a source of antagonists of Wnt, BMP and Nodal signalling (Arkell and Tam, 2012). It is thought that the action of the AVE positions or restricts the PS to the opposite end of the epiblast (Perea-Gomez et al., 2004; Perea-Gómez, 2014; Rivera-Pérez and Magnuson, 2005). Our results raise questions about the actual role of the AVE, since they show that a stable axis, as reflected by localised expression of Bra, Sox17 and FoxA2, can be initiated without external influences. In our experiments the signals are ubiquitous and so the symmetry-breaking event must be intrinsic to the aggregates, raising the possibility that a similar spontaneous event takes place in the embryo. This conclusion is at odds with the large body of experimental evidence suggesting that the anteroposterior axis requires a sequence of interactions between extraembryonic and embryonic tissues (Rossant and Tam, 2009).

One way to reconcile our observations with those of the genetic analysis of early development would be to entertain the possibility that the function of the AVE is not to break the symmetry of the embryo but rather to ensure that an event that can happen spontaneously has a reproducible outcome, i.e. the AVE ensures the maintenance of a region primed for anterior neural development at the opposite pole to that of the PS and, more importantly, endows this region with an anterior neural fate potential (Albazerchi and Stern, 2007; Bertocchini and Stern, 2002). This suggests that it is possible to uncouple symmetry breaking and anterior neural specification. The latter requires suppression of Nodal, BMP and Wnt signalling (Andoniadou and Martinez-Barbera, 2013; Stern, 2005) and this, in terms of patterning anterior and posterior domains, can only be achieved by a localised source which, in the embryo, is provided by the AVE. Consistent with this, aggregates maintained in N2B27, or in N2B27 in the presence of BMP inhibitors, for the most part remain symmetrical and express a neural fate, probably mimicking the specification of anterior neural fate under these conditions (Eiraku et al., 2011). In the future it will be interesting to provide localised inhibition of BMP and Nodal in aggregates exposed to Act, BMP and Chi to try to obtain both anterior neural and mesendodermal fates in the same group of cells. However, this only provides a partial explanation, as embryos lacking a VE develop symmetrically (Perea-Gómez et al., 1999; Waldrip et al., 1998), suggesting that our experimental conditions might be generating a situation that does not occur in the embryo. One explanation is that, in addition to maintaining a proneuroectodermal region, a key function of the AVE is to bias a spontaneous symmetry-breaking event that is intrinsic to the epiblast. Our experiments might be creating these imbalances by an excess of specific signals in the medium, a hypothesis that will be of interest to test in further experiments.

The events that lead to symmetry breaking remain out of the scope of this work; however, our observations provide some hints as to their constraints. There is clearly a defined length scale to the process, as only aggregates of a certain size undergo the unique event. The symmetry-breaking event must contain an activating and an inhibitory component that are linked, i.e. once the process has started it can inhibit itself within a certain length scale to make the process unique (Meinhardt, 2012). In support of this suggestion, there is evidence for the potential to generate multiple PSs or axes from a single embryo in mouse (Merrill et al., 2004; Perea-Gomez et al., 2002) and chicken (Bertocchini and Stern, 2002; Bertocchini et al., 2004) but only one emerges in the embryo. The possibility that limitation of signalling range plays a role can be gauged in our experiments, which show that persistent signalling can give rise to multiple patterning foci.

Finally, and in the context of symmetry breaking, our experimental system underpins a well-known connection between Wnt signalling and axial elongation (Martin and Kimelman, 2009; Petersen and Reddien, 2009) and provides an opportunity to probe into its mechanism.

### Gastrulation in culture?

In the mouse embryo, one of the consequences of the formation of an anteroposterior axis is the localisation of the start of gastrulation to the posterior proximal region, a process that will generate the primordia for the endoderm and the mesoderm as well as reveal the axial organisation of the embryo (Nowotschin and Hadjantonakis, 2010; Ramkumar and Anderson, 2011; Tam and Gad, 2004). The start of this process is manifest in the localisation of the expression of BMP, Nodal and Wnt3 to this region and, more significantly, of Bra to the emergent PS (Herrmann, 1991; Pfister et al., 2007). A central feature of this structure is an epithelial-to-mesenchymal transition (EMT), which, under the control of specific signals, leads to a germ layer-specific behaviour: in the endoderm, cells re-epithelialise (Burtscher and Lickert, 2009; Kwon et al., 2008; Lewis and Tam, 2006), whereas in the mesoderm they become highly mesenchymal (Nakaya and Sheng, 2008). As a result of these movements the three germ layers are distributed relative to each other. We observe related behaviours when our aggregates are exposed to Act, BMP and, in particular, Wnt/β-catenin. On its own, Act treatment elicits the expression of the endodermal marker Sox17 in a group of cells that express E-cadherin and form coherent epithelial groups on the outer edges of the aggregate, as they do in the embryo. By contrast, BMP and especially Wnt/β-catenin favour the extrusion of cells from a domain that expresses Bra and TBX6, a gene associated with paraxial mesoderm (Chapman et al., 1996). These observations suggest that our culture system recapitulates some of the features of gastrulation, even though the behaviour of the mesodermal-like cells is the reverse of that in the embryo, where cells move inward rather than outward. It is likely that this topological switch reflects the architecture of the aggregates.

**Fig. 9.**
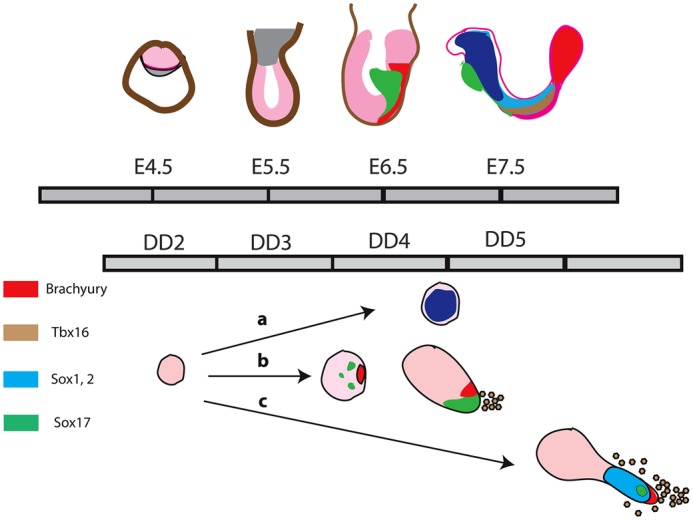
**Comparison of events in embryos and aggregates.** (Top) Timeline of embryogenesis, with the illustrated stages acting as landmarks. (Bottom) A representation of the behaviour of aggregates exposed to different signalling environments over the indicated periods of differentiation, as inferred from our experiments labelled here as a, b and c. We propose that the third day of differentiation of the aggregates is equivalent to the E5.5-6.0 postimplantation epiblast. DD, day of aggregate differentiation. The dark blue shading indicates anterior Sox1 expression.

At the end of gastrulation, amniote embryos undergo a process of axial extension that generates the spinal cord and the paraxial mesoderm from a population of stem-like cells located in the distal end of the embryo (Kondoh and Takemoto, 2012; Wilson et al., 2009). This process relies on Wnt signalling and a localised source of Bra expression at the tip of the extension (reviewed by Wilson et al., 2009). We observe that transient exposure of the aggregates to Chi is able to elicit this structure (see also Turner et al., 2014a). Furthermore, in some of these aggregates we observe endoderm embedded in this tissue near its surface, a situation that, once again, mimics the embryo (Fig. 9).

We note that, independent of the geometry, the sequence and timing of the events that we observe are reproducible and can be related to events in the embryo (Fig. 9). Thus, day 3 seems to be similar to E5.5-6.5 as it is at this time that we observe the cell extrusion and intrusion, with the elongation starting sometime during day 4, which would thus be homologous to E7.5 in the embryo. Altogether, these observations further emphasize the similarity between the processes that we have uncovered here and the events in the embryo. The movements are related to those of cells in gastrulating embryos and for this reason we term these aggregates ‘gastruloids’.

### Final consideration and prospective uses of gastruloids

A somewhat surprising aspect of the gastruloids is that they allow the uncoupling of processes that in the embryo are tightly linked, such as specification of the anteroposterior axis and anterior neural development or endoderm specification and axial elongation. This could be construed to mean that, on the whole, the aggregates are not reflecting the situation *in vivo*. This contention could be underlined by the differential relative topology of the movements of endodermal and mesodermal cells in the aggregates. However, we believe that gastruloids reflect embryonic events and that they do so in the same manner as the emergence of eye cups (Nakano et al., 2012) and cerebroids (Lancaster et al., 2013) and offer an additional experimental system with which to explore the mechanisms of self-organisation processes in cellular ensembles. Furthermore, we believe that the deconstruction of developmental events achieved here and its comparison with the events in embryos will allow a detailed mechanistic analysis of processes that, like gastrulation and axial extension, have significant mechanical and geometrical inputs that make them difficult to study *in vivo*. Naturally, the conclusions from this work will ultimately have to be tested in embryos, but this should not deter their use for analytical purposes. We have begun to do this by probing into the mechanisms of axial elongation (Turner et al., 2014a).

## MATERIALS AND METHODS

### Tissue culture, FACS, immunofluorescence and confocal microscopy

Routine tissue culture, FACS analysis, immunofluorescence and confocal microscopy were performed as described previously (Faunes et al., 2013; Kalmar et al., 2009; Turner et al., 2014a,b,c). Primary antibodies used for immunofluorescence were: goat anti-Bra (Santa Cruz Biotechnology, sc-17743; 1:200), rat anti-E-Cadherin (Takara, M108; 1:200), goat anti-Sox17 (R&D Systems, AF1924; 1:500) and goat anti-FoxA2 (Santa Cruz Biotechnology, sc-6554; 1:500). Alexa-conjugated secondary antibodies were from Invitrogen and were used at 1:500 dilution. Hoechst 3342 (Invitrogen) stained the nuclei and was used at 1:1000 dilution.

### Cell lines

The cell lines used are E14-Tg2A, Sox1::GFP (Ying et al., 2003), Sox17::GFP (Niakan et al., 2010), Bra::GFP (Fehling et al., 2003), the Wnt/β-catenin transcriptional reporter TCF/LEF::mCherry (Faunes et al., 2013; Ferrer-Vaquer et al., 2010), TBX6::EYFP (this is a knock-in into the *Tbx6* locus; A.-K.H. and S.N.) and CAG::GPI-GFP (referred to hereafter as GPI-GFP) (Rhee et al., 2006).

### Aggregate culture and imaging

A detailed protocol for the growth of the aggregates, with trouble-shooting, is provided elsewhere (Baillie-Johnson et al., 2014). Images in Fig. 1 were generated by manipulating the brightness and contrast of pictures of the aggregates in addition to edge detection; the outlines were enhanced manually through tracing. The original unprocessed images of the aggregates are provided in supplementary material Fig. S1G,H. N2B27 (NDiff) was sourced from StemCells (USA) and tissue culture slides for monolayer imaging were obtained from Ibidi (Germany). All experimental conditions were repeated at least twice.

## Supplementary Material

Supplementary Material
