# Supplementary material for: Symmetry breaking, germ layer specification and axial organisation in aggregates of mouse embryonic stem cells
Source: Development. 2014 Nov 15;141(22):4231–42. doi: 10.1242/dev.113001 (PMC4302915; doi:10.1242/dev.113001)
Supplement: Supplementary Material [file supp_141_22_4231__index.html]

Supplementary Material 

# Symmetry breaking, germ layer specification and axial organisation in aggregates of mouse embryonic stem cells

## DEV113001 Supplementary Material

**Files in this Data Supplement:**

- Supplementary Material
