# Supplementary material for: Symmetry breaking, germ layer specification and axial organisation in aggregates of mouse embryonic stem cells
Source: Development. 2014 Nov 15;141(22):4231–42. doi: 10.1242/dev.113001 (PMC4302915; doi:10.1242/dev.113001)
Supplement: Supplementary Material [file supp_141.22.4231_DEV113001supp.pdf]

**Supplementary Figure Legends**

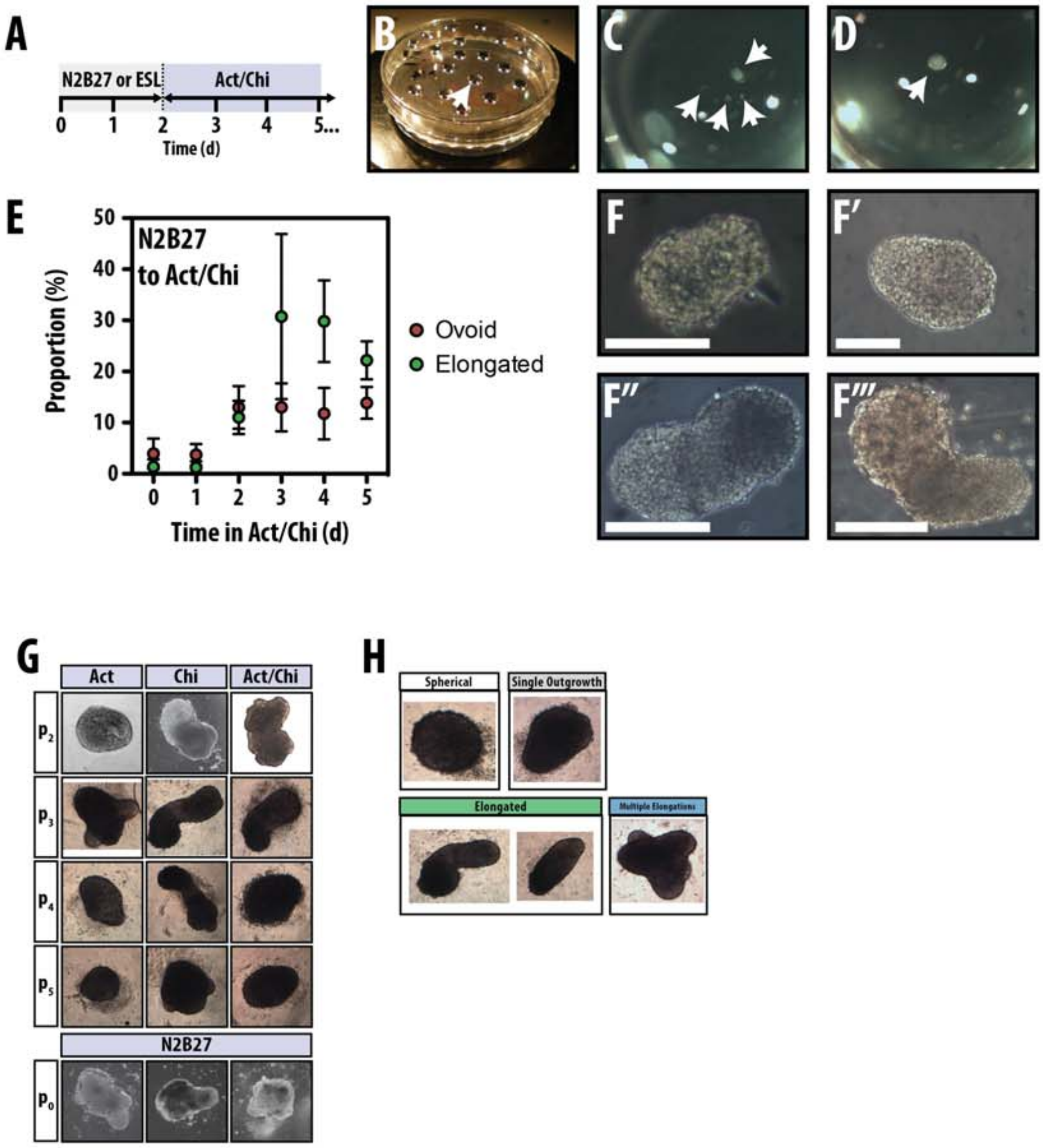


# Figure S1

**Figure S1.** Elongation of aggregates from mouse ESCs. (A) Schematic representation of differentiation time-course; HD: aggregates forming in hanging-drops; Act/Chi: Medium condition of 100 ng/ml Act + 3 μM Chi. (B) Hanging drops of ~200 mESCs per 40 μl drop. (C) mESCs in HD phase grown in Serum with LIF or (D) N2B27 only. (E) Proportion of cells displaying elongated morphologies following HD phase of N2B27. Each point represents the median from two independent experiments and (F-F‴) Representative images of elongating cells from (E) after 1 (F), 3 (F′), 6 (F″) and 11 (F‴) days Act/Chi. Arrows in (C) and (D) indicate individual aggregates. (G) Original, unprocessed images used to generate the cartoon renderings of the typical aggregate morphologies in Fig. 1B. Maximum elongation was observed following pulsed treatment within the day 2-3 time-frame. (H) Original, unprocessed images of aggregates used to generate the examples of morphologies used in Fig. 1C. See materials and methods section for explanation of rendering process. Images not to scale. Scale bar: 100 μm.

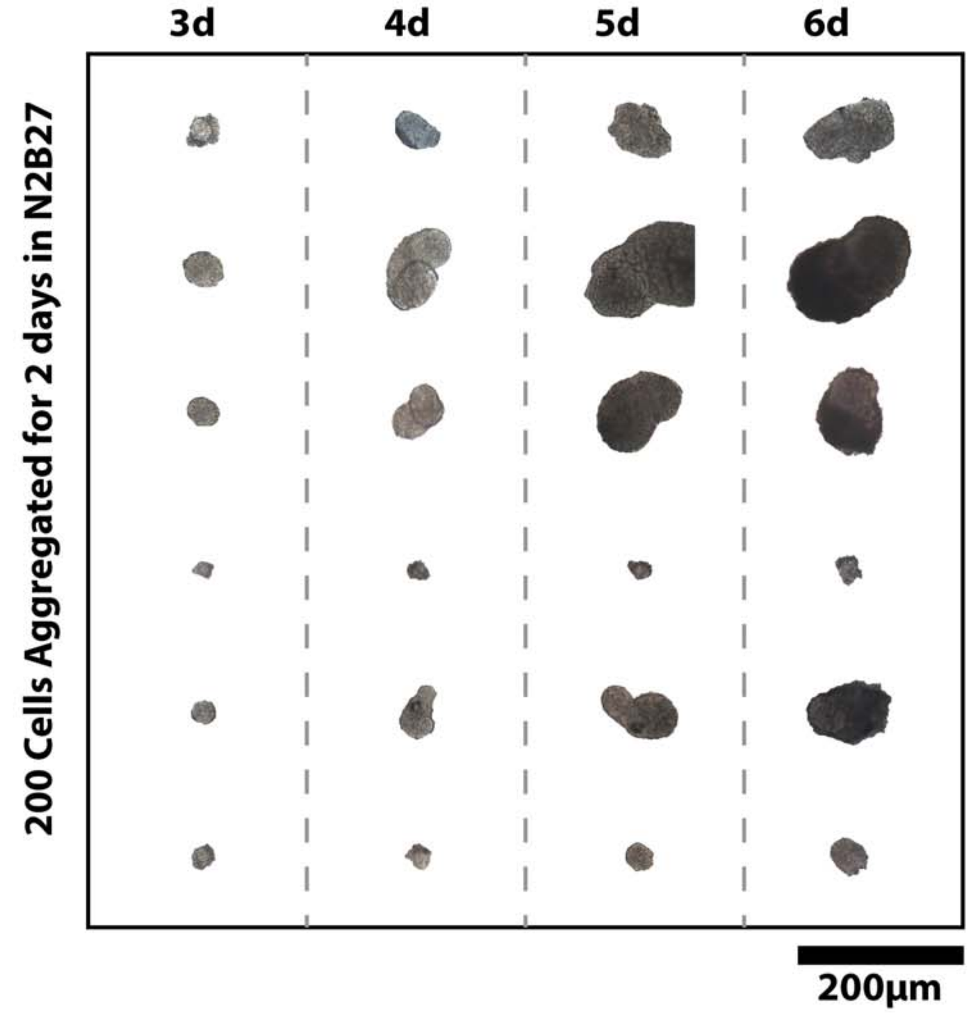


Figure S2A

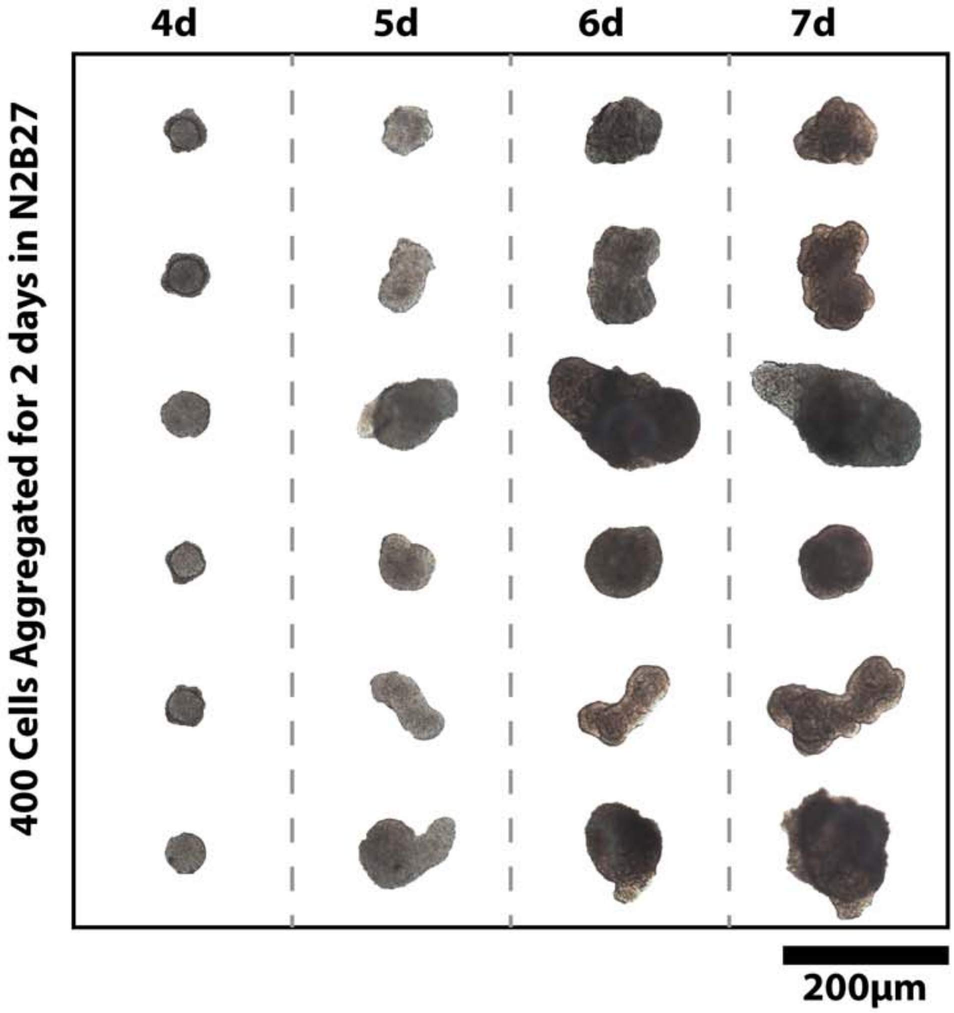


**Figure S2B**

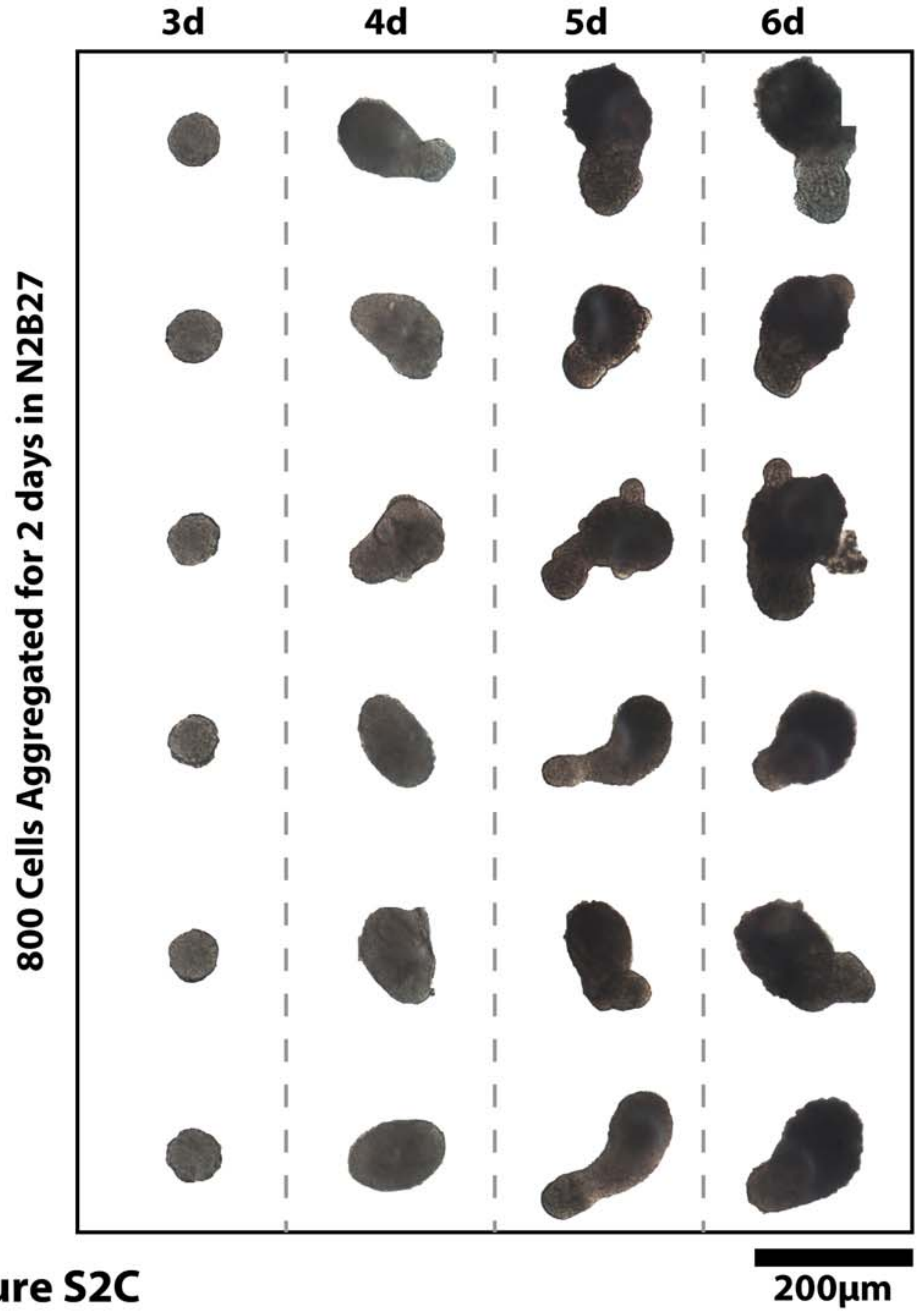


Figure S2C

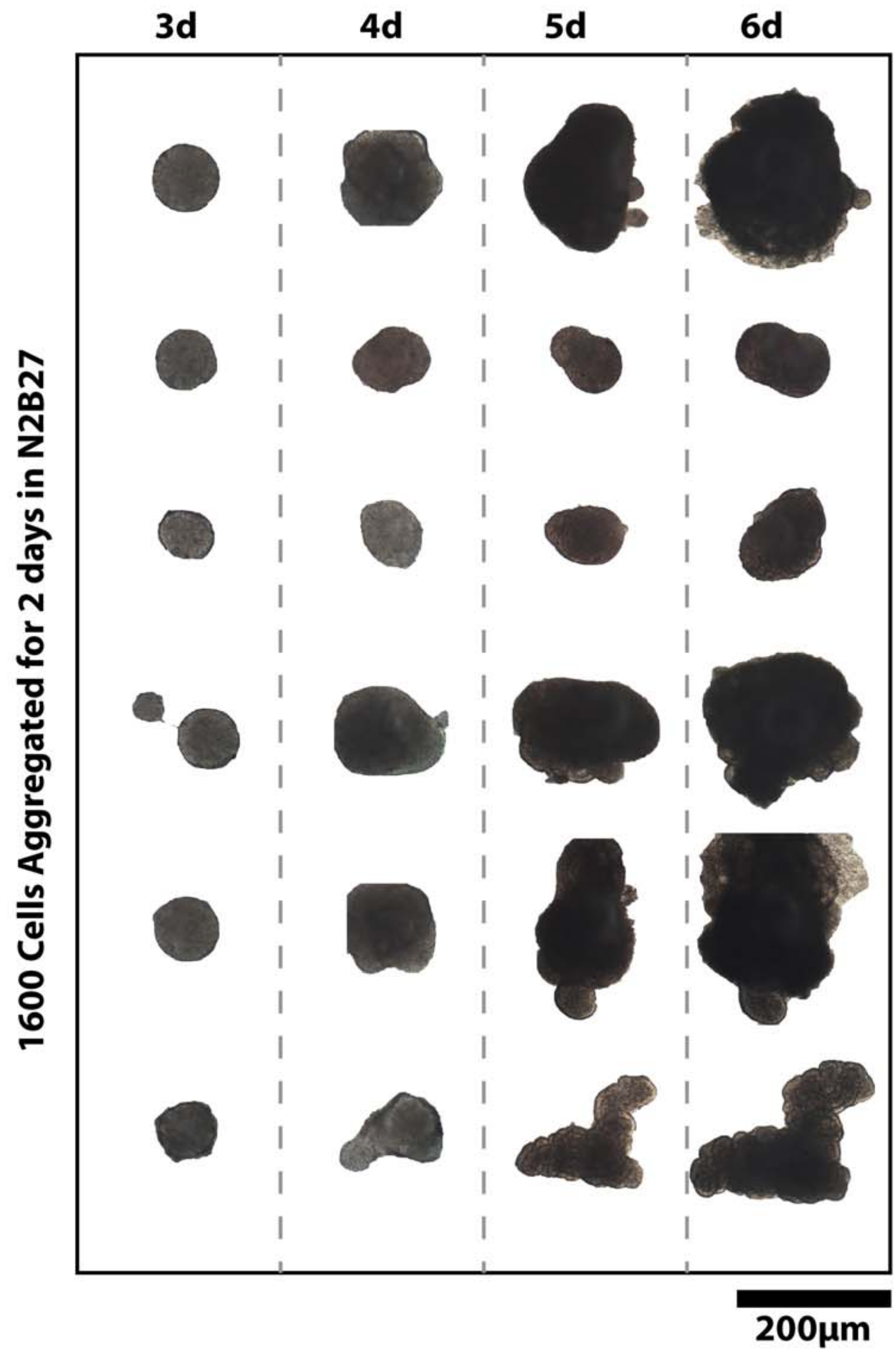


Figure S2D

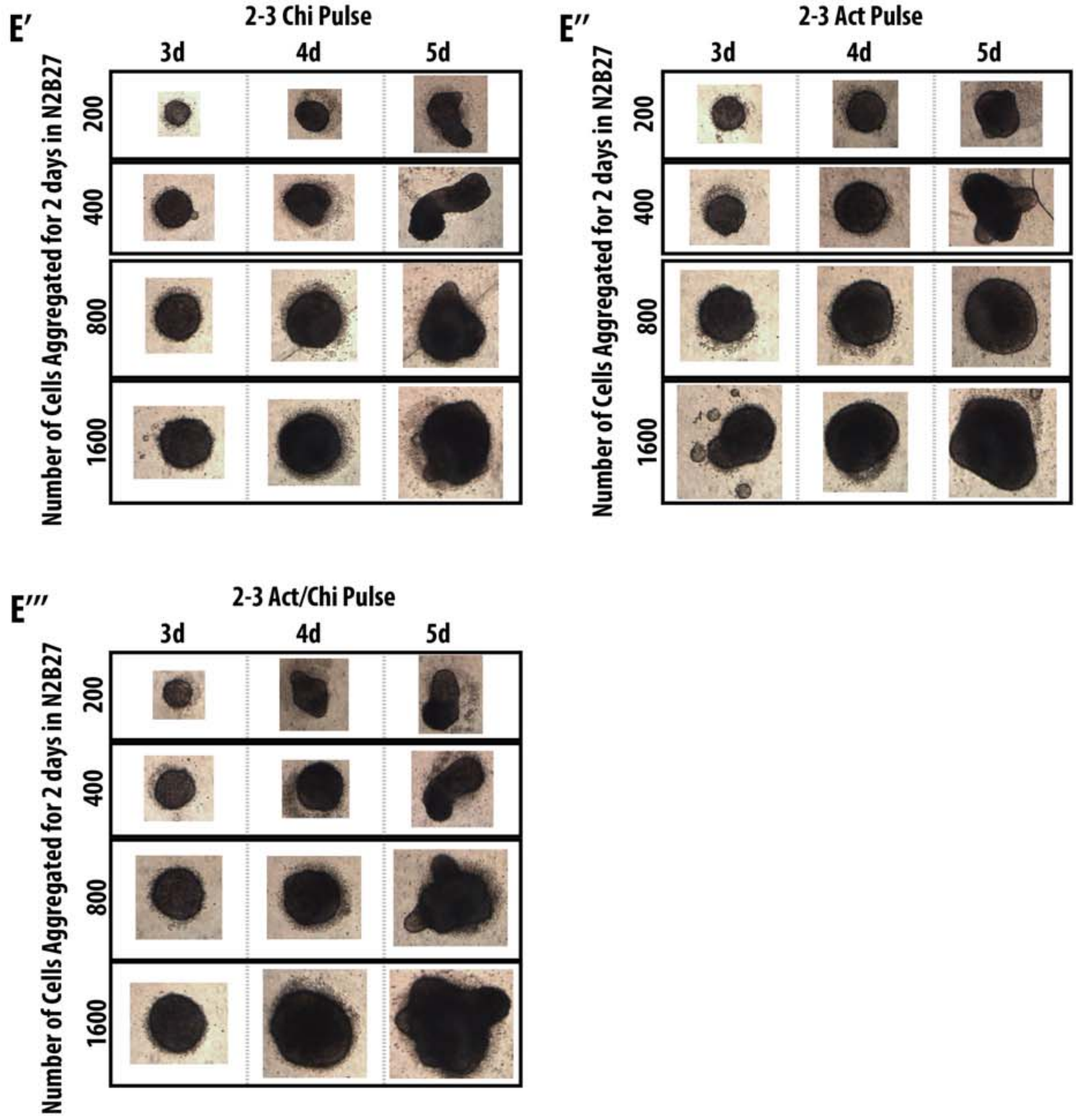


# Figure S2E

**Figure S2**. A comparative analysis of exposure time, signalling and initial cell density on aggregate formation. (A-D) Aggregates formed from 200 (A), 400 (B), 800 (C) and 1600 (D) cells and imaged daily from day 3 to 6.

Six representative examples per condition are shown. Scale bar: 200 µm. (E) Aggregates formed using different initial plating densities (200, 400, 800 and 1600 cells per well) were cultured in N2B27 with a 24 h pulse on day 2-3 of Chi (E′), Act (E″) or Act/Chi (E‴). Representative aggregates are shown for each condition plating density; the same aggregate was followed throughout the time-course. See Fig. 2A (protocol P3) for stimulation protocol. Aggregates in (E) not to scale.

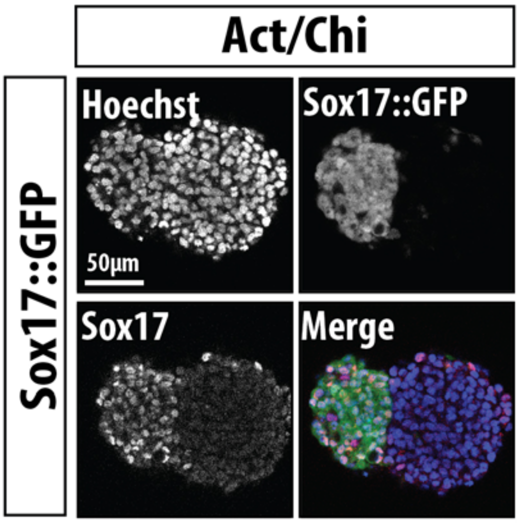


# Figure S3

**Figure S3.** Sox17::GFP faithfully reports the localisation of Sox17. Sox17::GFP cells exposed to continuous Act/Chi after 2 days N2B27 were fixed and stained for Sox17. Confocal imaging confirms the faithful reporting of *Sox17* gene expression by the Sox17::GFP fluorescent reporter. Scale bar: 50 µm. Hoechst was used to stain the nuclei.

**Supplementary Movie Legends**

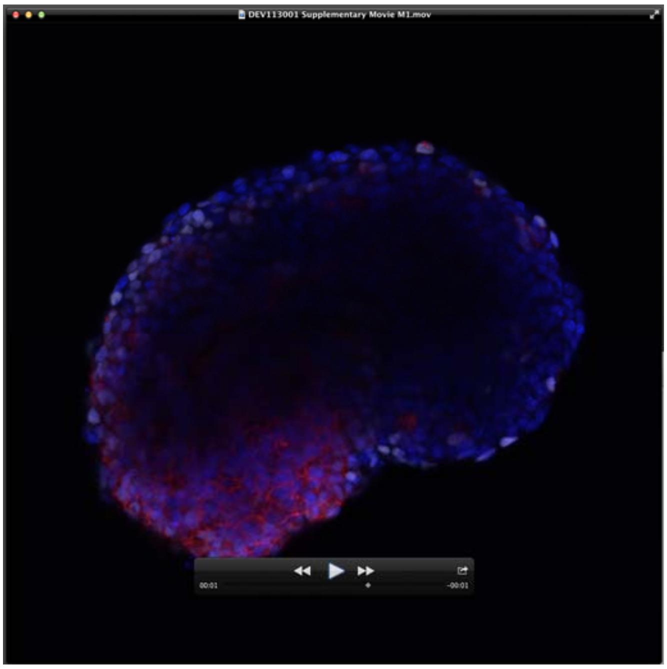


**Movie 1.** Sections through the aggregate shown in Fig. 3B and B'. Hoechst, E-Cadherin and Sox17 are labelled in blue, red and white, respectively.

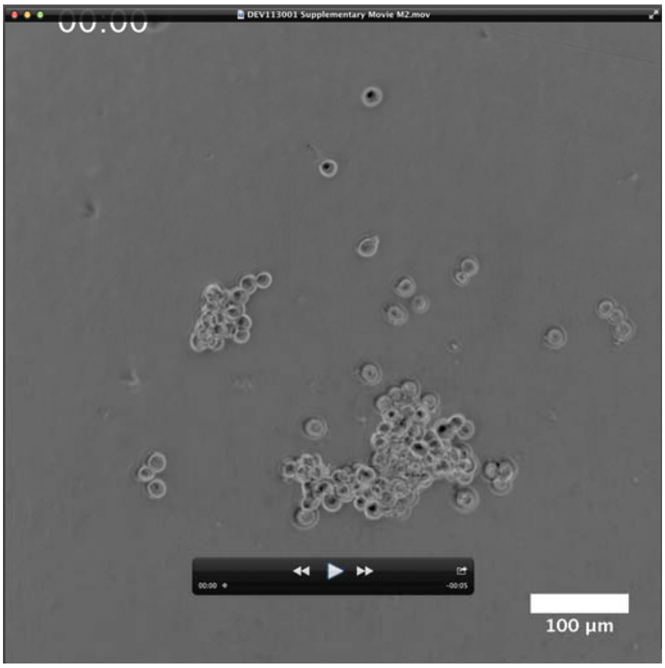


**Movie 2**. Aggregation of ES cells within the first 48 h of suspension culture. A suspension of mouse ES cells in N2B27 were plated in 96-well plates as described (see Materials and Methods; and Baillie-Johnson et al., 2014) and imaged for 48 h. Over time, individual or small clusters of ES cells begin to aggregate at the bottom of the well (refer to Fig. 5A).

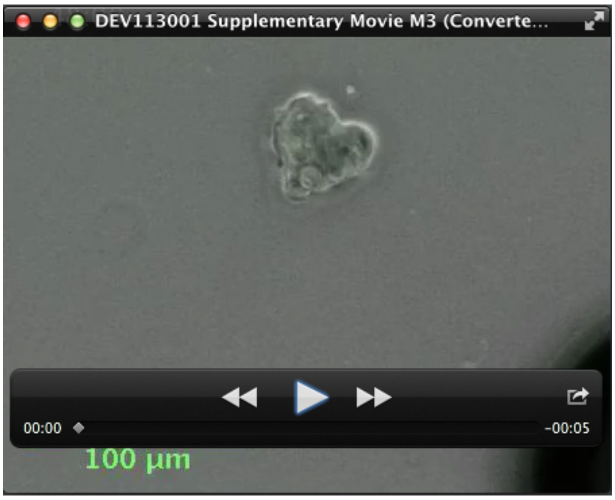


**Movie 3.** Emergence Sox17::GFP expression following addition of Act and Chi. Aggregated Sox17::GFP ES cell were imaged for ~73 h in N2B27 supplemented with Act and Chi. Note how the expression of Sox17::GFP is heterogeneously expressed before a regionalised expression pattern is adopted (see Fig. 5B).

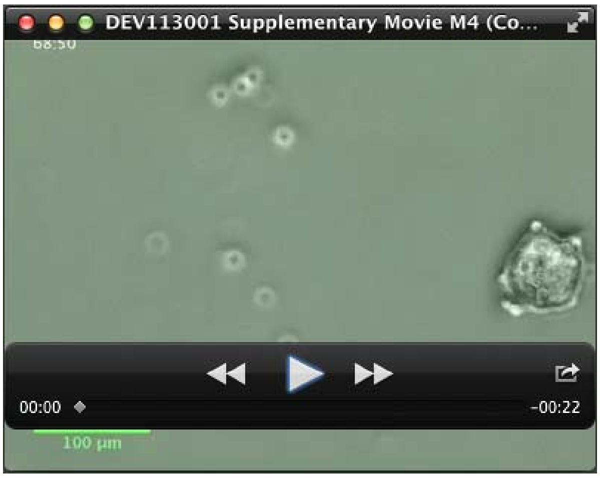


**Movie 4**. Generation of polarised expression of Sox17::GFP. Polarised expression of Sox17::GFP becomes more pronounced as the aggregate increases in size throughout time (see Fig. 5C).

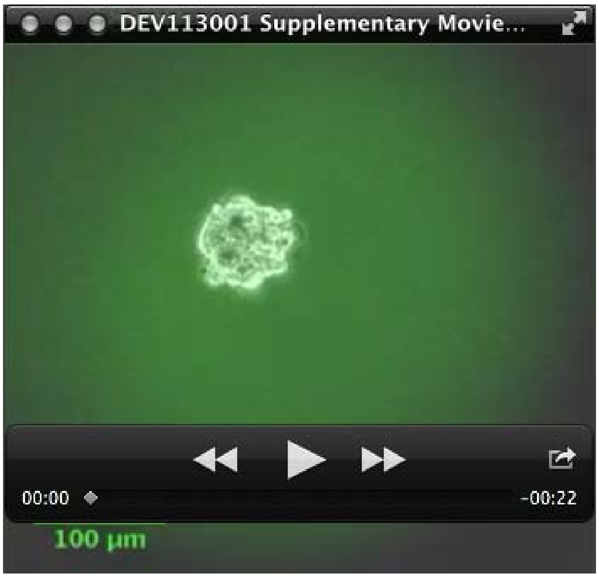


**Movie 5.** Formation of polarised Bra::GFP expression in aggregated ES cells. Aggregates of Bra::GFP ES cells in N2B27 were transferred to Act and Chi and filmed for 120 h. Initially, Bra::GFP is expressed throughout the whole aggregate before the down-regulation in regions that will not form the extensions (see Fig. 5E).

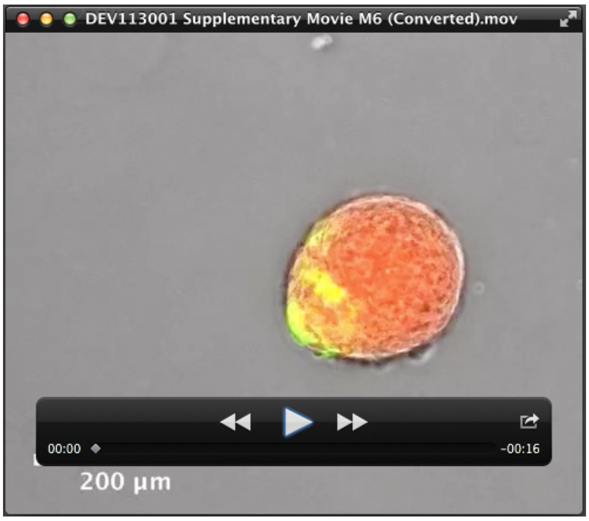
DEV113001 Supplementary Movie M6 (Converted).mov
00:00
-00:16
200 µm

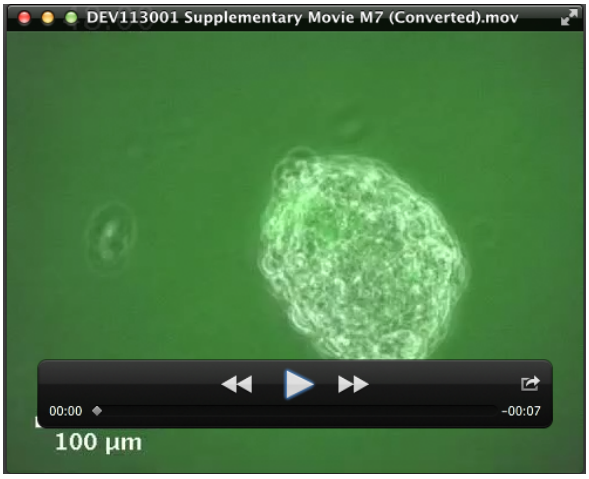
DEV113001 Supplementary Movie M7 (Converted).mov
00:00
-00:07
100 µm

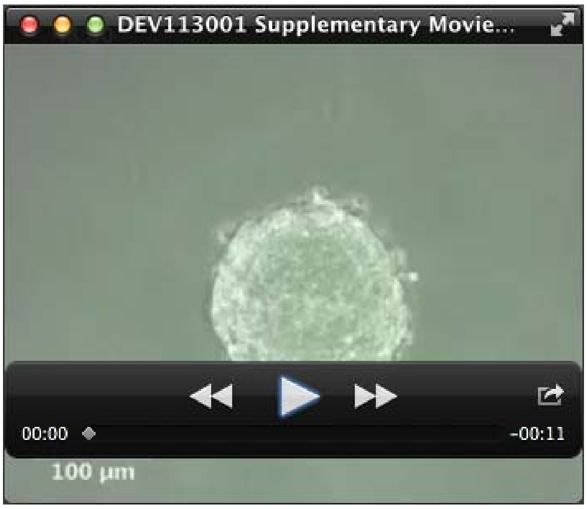
DEV113001 Supplementary Movie...
00:00
-00:11
100 µm

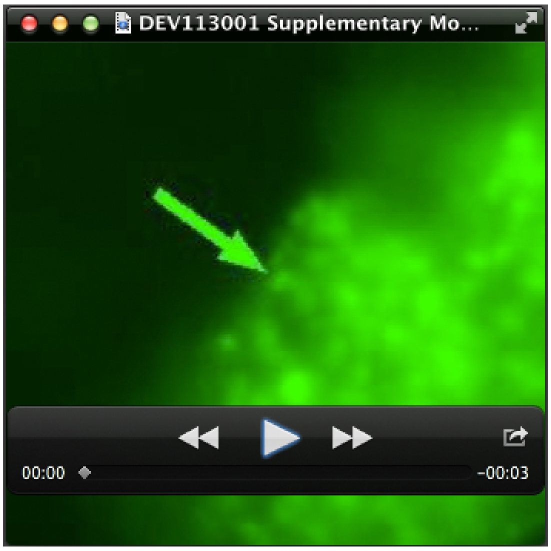


**Movies 6-9.** Gastrulation-like movements in aggregates. Cell extrusion and intra-aggregate movement in Sox17::GFP (Movie 6), Bra::GFP (Movie 7) and Tbx6::EYFP (Movies 8,9). Aggregates were treated as in Fig. 8 indicated and the stills correspond to the indicated time points from the associated movies (see Movies 6-9).

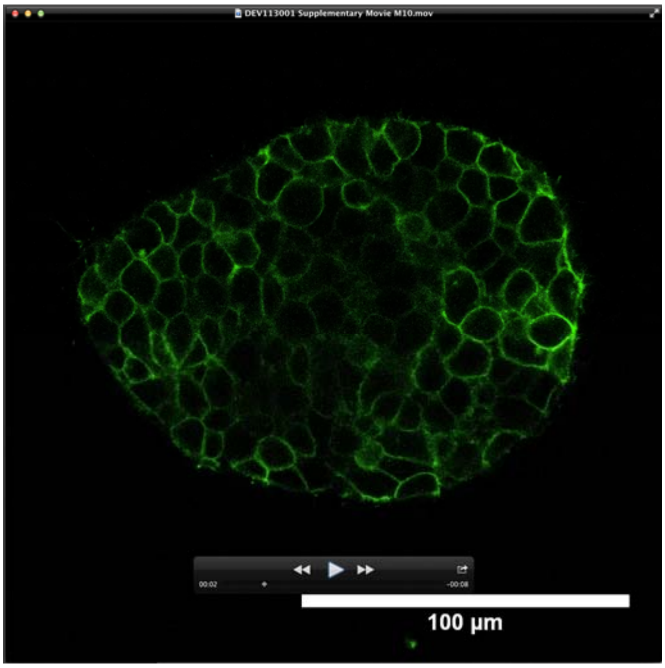


**Movie 10**. Polarised cell blebbing in an aggregate. Aggregates of GPI-GFP ES cells were transferred to Act and Chi conditions on day 3 and were imaged over 2 h with confocal microscopy.
